# Oral Lichen Planus in a Patient With a Thymoma: A Rare Finding

**DOI:** 10.7759/cureus.17376

**Published:** 2021-08-23

**Authors:** EB Robson Gubod, Anand Ramanathan, Zuraiza Mohamad Zaini, Saman Warnakulasuriya

**Affiliations:** 1 Oral & Maxillofacial Clinical Sciences, Faculty of Dentistry, University of Malaya, Kuala Lumpur, MYS; 2 Oral Cancer Research & Coordinating Centre, Faculty of Dentistry, University of Malaya, Kuala Lumpur, MYS; 3 Faculty of Dentistry, Oral & Craniofacial Sciences, King's College London, WHO Collaborating Centre for Oral Cancer, London, GBR

**Keywords:** oral lichen planus, thymoma, pure red cell aplasia, topical steroids, cyclosporin, management

## Abstract

We present a rare case of ulcerative oral lichen planus that was associated with a thymoma discovered during the management phase, seven months after the initial diagnosis of oral lichen planus. Thymectomy was performed and investigations revealed pure red cell aplasia. Although rare, the association of a thymoma should be considered in recording the medical history of patients presenting with oral lichen planus.

## Introduction

Oral lichen planus (OLP) is a chronic inflammatory disease of the oral mucosa which affects 1 - 2% of the population [1]. Thymoma is a neoplasm of the thymus epithelial cells, which is commonly associated with autoimmune diseases such as myasthenia gravis and systemic lupus erythematosus [2]. OLP associated with thymoma is rarely seen in clinical practice. Here we report a case of lichen planus associated with thymoma which was discovered seven months after the diagnosis of OLP.

## Case presentation

A 45-year-old Chinese man presented with the complaint of pain and burning sensation in the mouth associated with hot and spicy food consumption for an approximately one-month duration. At presentation, he had no known systemic illness and was not on any medications. He occasionally consumed alcohol and had never smoked. There was also no history of usage of a new toothpaste, new mouthwash, and/or undergoing dental restoration work before symptoms appeared. At the initial visit, there were no skin, genital, scalp lesions, or nail abnormalities. He did not have any ptosis. 

**Figure 1:**
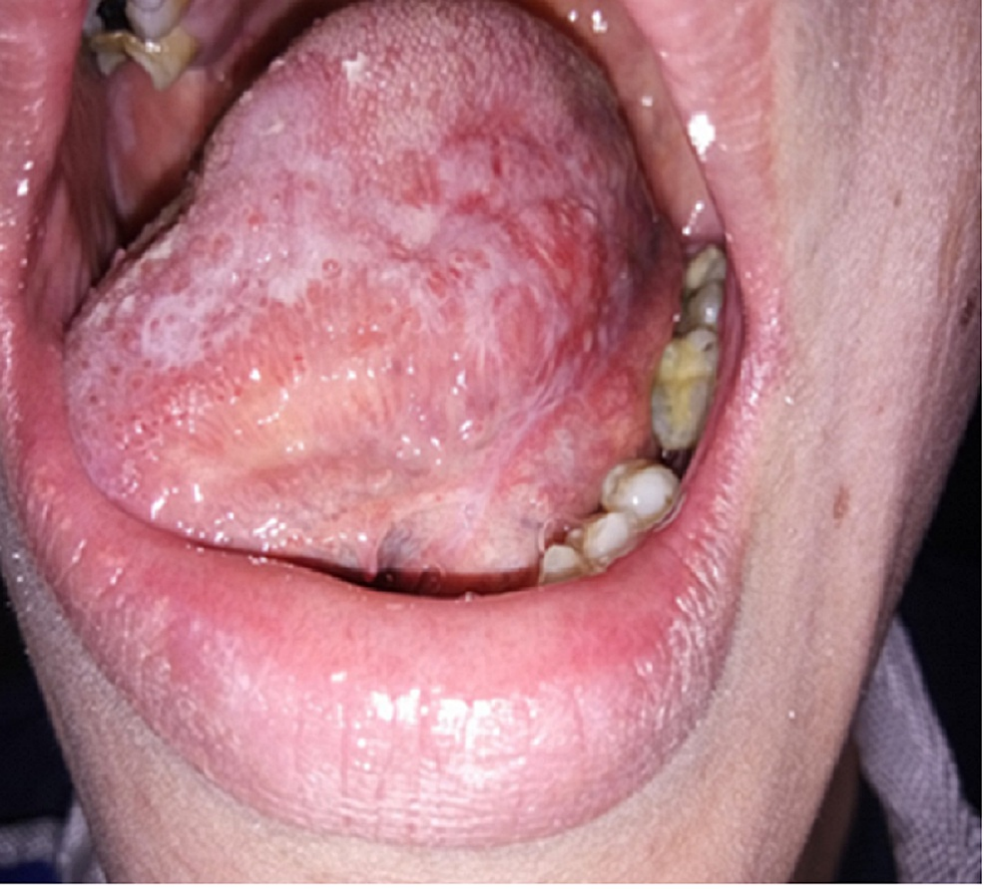
Intraoral photograph shows the left lateral border of tongue and dorsum having areas of erythema and white striations on initial presentation.

On examination, the patient presented with symmetrical, white interlacing striations with erythema and superficial ulcerations on bilateral buccal mucosal surfaces. Erythema and ulceration were noted on the left lateral border of the tongue along with keratotic plaques on the dorsal surface of the tongue (Figure 1). No induration was noted. Other mucosae were unaffected. Several dental restorations composed of glass ionomer cement and composite resin were noted adjacent to the lichenoid lesions on the buccal mucosa. A provisional diagnosis of OLP/ oral lichenoid reaction was established.

An incisional biopsy from the left lateral border of the tongue was performed and sent for routine histopathological examination and direct immunofluorescence. Histopathological findings confirmed OLP. Direct immunofluorescence was negative for fibrinogen, complement protein C3, immunoglobulin (Ig) A, IgG, and IgM, excluding any other immunological mucosal disorder. A skin patch test was carried out using the European standard patch test kit (Chemotechnique diagnostics, Vellinge, Sweden) along with dental material screening series. The patient showed no hypersensitivity to any of the tested substances, including the dental materials.

After establishing the diagnosis of OLP, the patient was started on topical steroid therapy using topical dexamethasone - 1 mg/g (Dexaltin® oral paste). After initiation of topical steroid therapy, the patient’s symptoms improved, reporting less burning sensation. He was put on follow-up every two to four weeks interval for four months. During these follow-ups, the patient was mainly treated with topical corticosteroids (Dexaltin®). After four months of follow-up the patient failed to attend the clinic for a period of three months.

**Figure 2:**
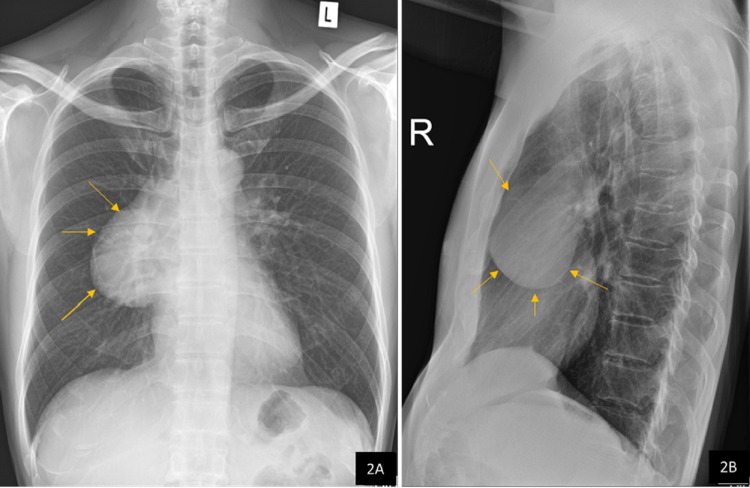
Chest radiographs (A) posteroanterior view and (B) lateral view show right paravertebral smooth convex mass overlying the hilum.

During this period the patient had felt extreme lethargy for few days and had sought treatment at a medical facility. On investigation his full blood count revealed severely decreased hemoglobin level which was 6.4 g/dl (normal range 13.0 - 18.0 g/dl); red cell count 2.2 x 10^12^/l (normal range 4.5 - 5.9 x 10^12^/l); hematocrit 19% (normal range 41 -53%). His chest radiograph revealed a mediastinal mass (Figure 2A, 2B) which was diagnosed as a thymoma.

**Figure 3:**
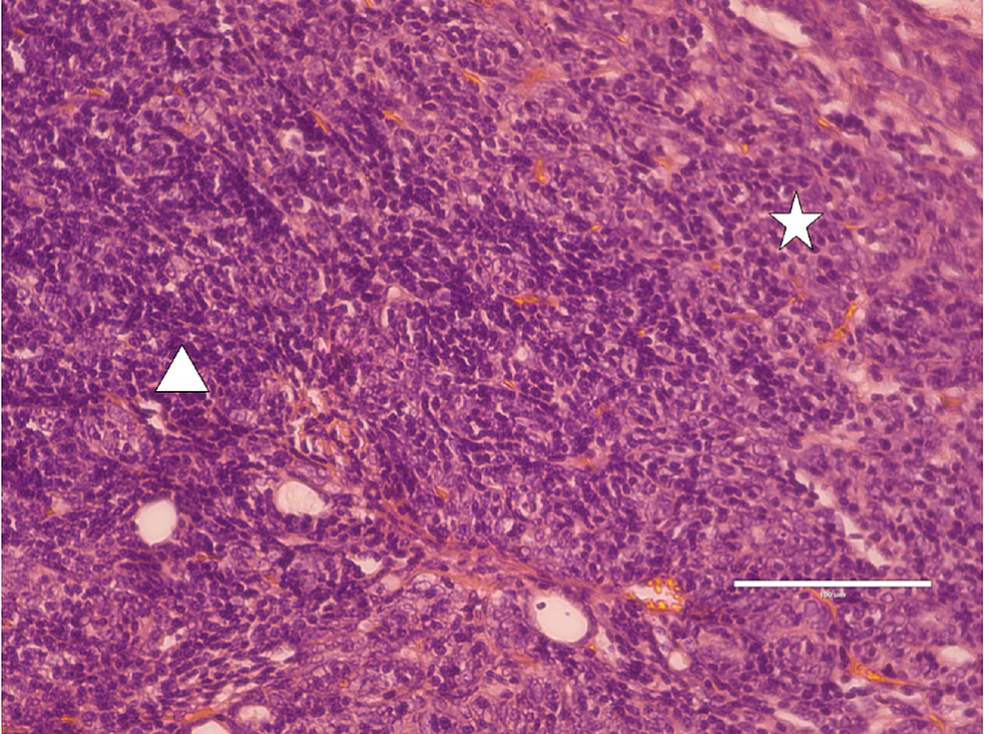
Photomicrograph shows a mixture of lymphocyte-poor type A (star) and lymphocyte-rich type B (triangle) components (H&E, x400). H&E - Hematoxylin and Eosin stains

His thymoma was treated by surgical excision. The pathology report revealed a mixture of lymphocyte-poor type A and lymphocyte-rich type B thymoma components. The type A component consisted of plump, short, spindle-shaped tumor cells, and type B presented with predominantly mature lymphocytes along with scattered polygonal cells with a vesicular nucleus (Figure 3). The tumor had invaded the capsule and extended into the peri-tumoral adipose tissue. It was diagnosed as type AB thymoma (WHO Classification), minimally invasive.

**Figure 4:**
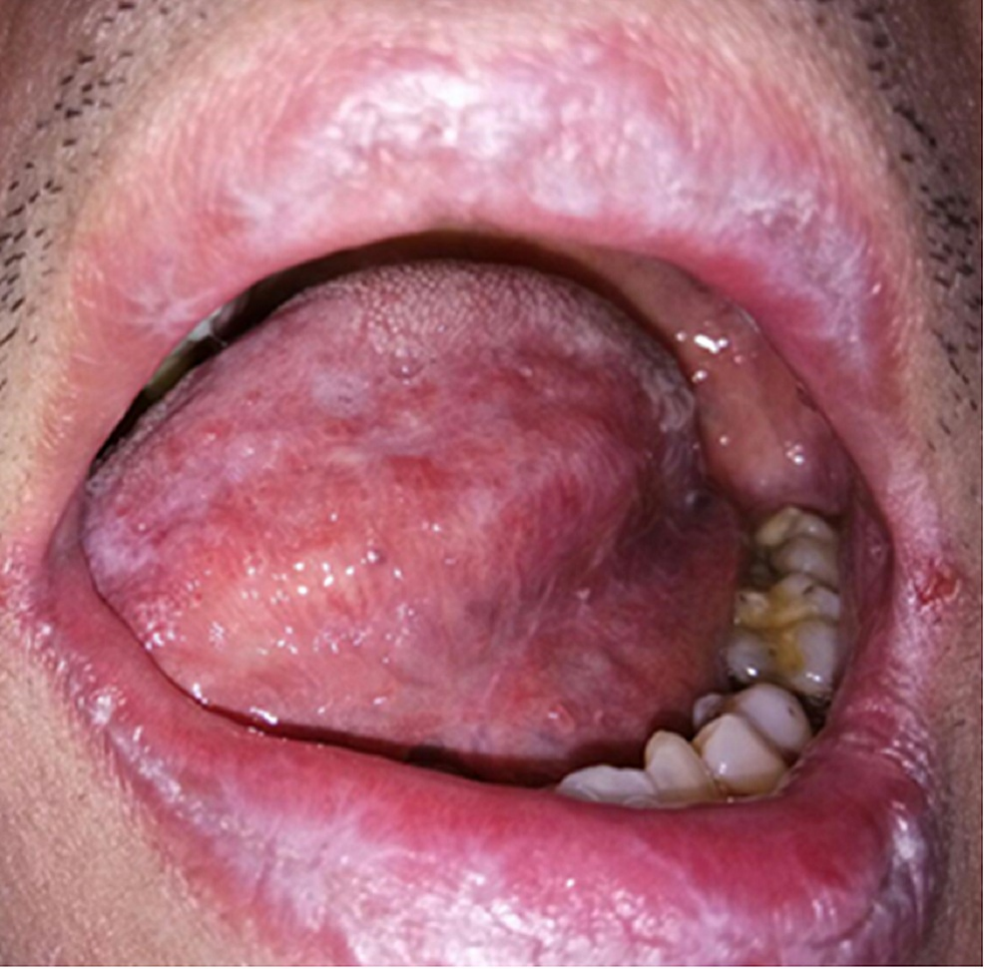
Intraoral photograph shows left lateral border of the tongue and dorsum having mild erythema after taking systemic cyclosporine.

**Figure 5:**
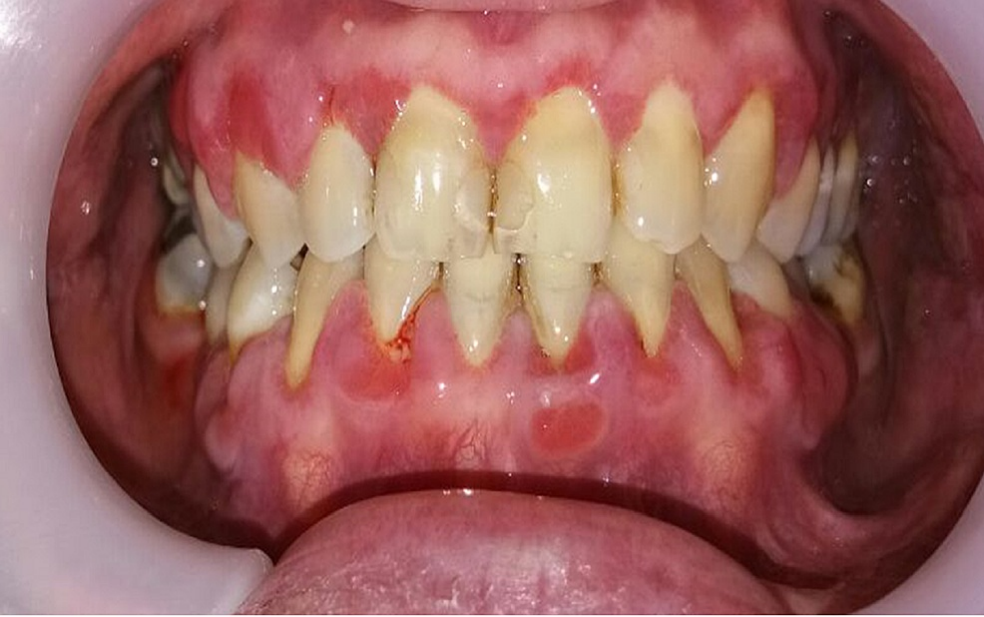
Intraoral photograph shows mild generalized gingival growth after taking systemic cyclosporine.

After thymoma removal, further investigation into his low blood cell counts, which were earlier observed in the hematology reports, was undertaken. A bone marrow trephine biopsy revealed markedly hypocellular marrow, with 5% cellularity, and the erythroid precursors were markedly reduced. The marrow trephine was interpreted as severely hypocellular marrow, consistent with pure red cell aplasia (PRCA). Thymoma-associated PRCA was established and the patient was treated with systemic cyclosporine 100 mg daily and while on cyclosporine therapy his OLP lesions resolved and became asymptomatic (Figure 4). However, he developed generalized gingival overgrowth which was due to cyclosporine (Figure 5). A diagnosis of drug-induced gingival overgrowth secondary to cyclosporine was established and he received non-surgical periodontal treatment for plaque control.

**Figure 6:**
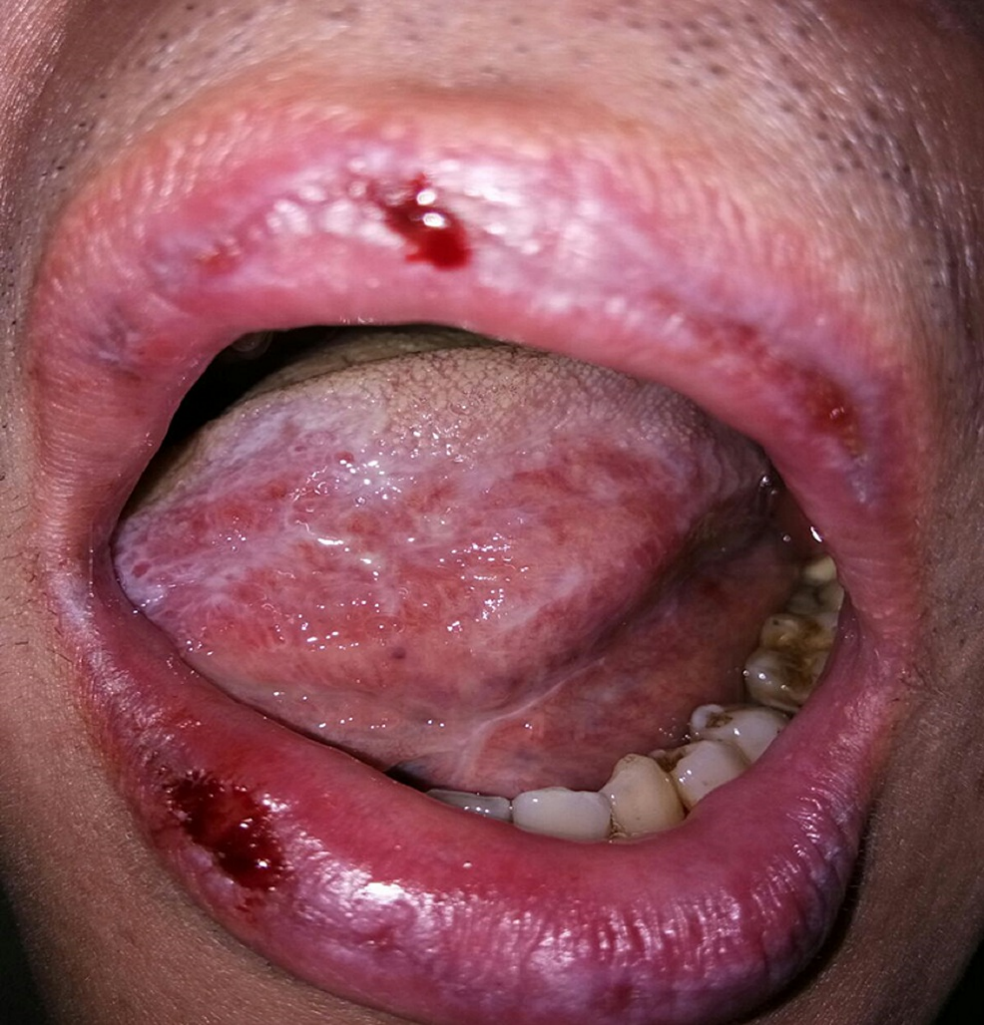
Intraoral photograph shows left lateral border of the tongue and dorsum having erythema and ulceration on the upper lip after reducing the dose of systemic cyclosporine.

Over three to six months, his cyclosporine dosage was gradually reduced and the gingival overgrowth started to resolve. However, his OLP recurred with white striations, and erythema and ulceration developed on his right and left buccal mucosa, the tongue, and lips (Figure 6).

**Figure 7:**
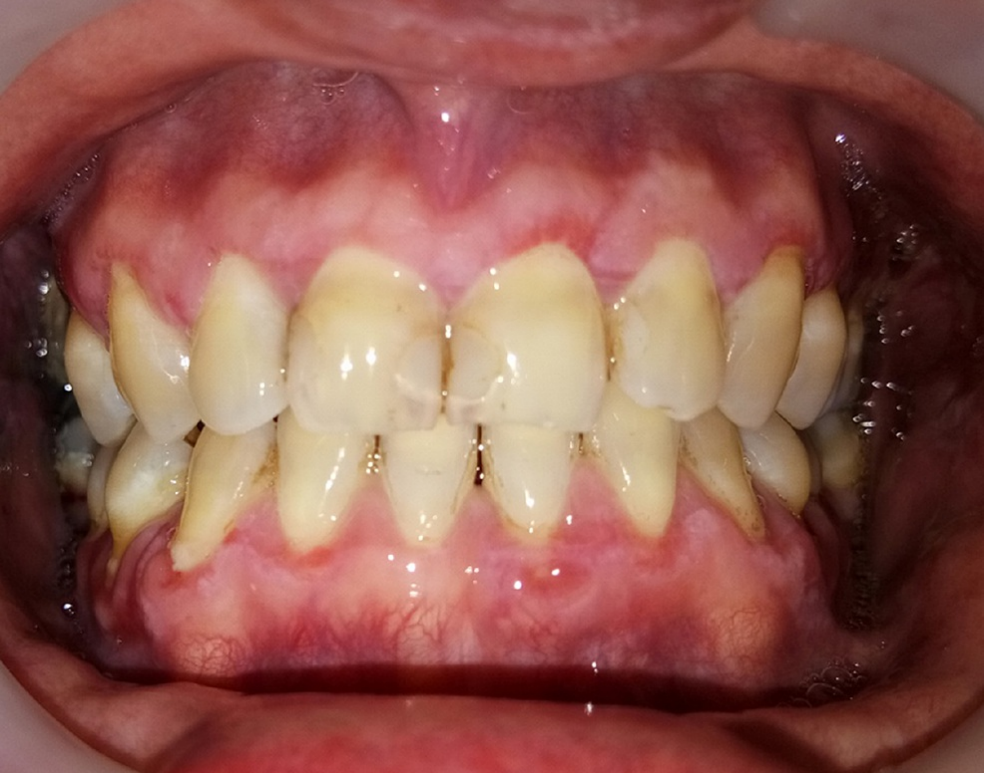
Intraoral photograph shows that the generalized gingival overgrowth has resolved following oral hygiene improvement and prophylaxis.

The patient was further managed with topical steroids and oral hygiene care to control his gingival overgrowth (Figure 7). 

After stopping the cyclosporine therapy, several flare-ups of his OLP were observed. The patient was managed mainly with topical corticosteroids (Dexaltin®), dexamethasone mouthwash, and intermittent short-term oral prednisolone 5 mg daily dose given for two weeks. The patient’s OLP lesion is overall moderately controlled with a topical corticosteroid (Dexaltin®). However, the patient responded well to systemic steroids, as the resolution of the lesion is seen after dosages of prednisolone 5 mg for two weeks.

## Discussion

Thymoma is a rare neoplasm of the thymic epithelial cells which commonly occurs in middle-aged individuals [3]. Approximately 30% of thymomas are asymptomatic, while non-specific symptoms such as cough, dyspnea, and superior vena cava syndrome are seen in another 30-40% of cases [3]. The etiology of thymoma is unknown, and most cases were detected incidentally during chest imaging for other reasons [4]. Thymomas are commonly associated with autoimmune disorders, the most common being myasthenia gravis [3]. Less frequently, in a small percentage of thymoma patients, hypogammaglobulinemia can develop to form Good Syndrome [5]. Good syndrome was first reported in 1954, it is characterized by adult-onset immunodeficiency, where the patient presents with thymoma and immunodeficiency [6]. The immunodeficiency is characterized by hypogammaglobulinemia and severely reduced or absent B-cells, CD4 lymphopenia, and CD8 lymphocytosis [5]. The association of Good Syndrome with erosive OLP, where patients presented with hypogammaglobulinemia, thymoma, and OLP has been reported [7].

Possible association of thymoma with OLP was first reported in the early 1970s [8]. Lichen planus had been reported in 0.4-1.1% of patients with thymoma [2]. Most of these patients presented with erosive OLP [2]. Paradoxically, lichen planus in a thymoma patient usually does not regress with thymectomy, which is the primary treatment of thymoma [2, 9]. OLP in patients with thymoma was reported to be resistant to treatment in most reported cases [10]. The pathogenesis of association between OLP and thymoma is unknown [10]. Miyagaki et al. proposed that abnormal regulation of lymphocytes within the thymus may cause OLP [11], and thymoma should be ruled out in patients with intractable lichen planus [10].

Pure red cell aplasia (PRCA) is a syndrome defined by normocytic anemia with severe reticulocytopenia and marked reduction or absence of erythroid precursor from the bone marrow [12]. PRCA can be congenital or acquired. Primary acquired PRCA is an autoimmune disorder in which the erythroid differentiation is interrupted by an immune mechanism, while secondary acquired PRCA is often associated with disorders such as collagen vascular disorders, lymphoproliferative disorders, parvovirus infections, and thymoma [12]. Thymoma is known as the disorder that has the highest association with secondary PRCA, and its discovery can precede the diagnosis of thymoma or can also be discovered after thymectomy [12], as in this case. As observed in this case, the management of thymoma-associated PRCA is chiefly by immunosuppressive therapy, where cyclosporine and corticosteroids are the immunosuppressive agents of choice [13]. 

The main management for thymoma consists of surgical removal or debulking [14], and the most important indicator for prognosis is the completeness of tumor resection [15]. Surgical removal can reduce the possibility of locally invasive growth and metastasis of thymomas; moreover, it usually has a favorable effect on associated disorders such as myasthenia gravis and PRCA [14].

## Conclusions

The purpose of this article is to report an unusual case of OLP which was recalcitrant to treatment. Although initial systemic evaluation did not reveal any associated medical condition, a thymoma was discovered as an occult condition during follow-up. This report provides further evidence that OLP can occur in association with thymoma. The medical-dental interface is important in managing oral mucosal diseases in association with dermatologists, and with specialists in immunology and endocrinology. This case report also highlights the importance of thorough medical history recording in patients presenting with recalcitrant OLP lesions. Based on the current evidence, diagnosis and follow-up of OLP are all the more important due to its potential malignant transformation to oral carcinoma and its association with several systemic diseases, a rare presentation being illustrated in this case.
